# Use of Cesarean Birth among Robson Groups 2 and 4 at Mizan-Tepi University Hospital, Ethiopia

**DOI:** 10.1155/2020/5620987

**Published:** 2020-09-04

**Authors:** Margo S. Harrison, Tewodros Liyew, Ephrem Kirub, Biruk Teshome, Andrea Jimenez-Zambrano, Margaret Muldrow, Teklemariam Yarinbab

**Affiliations:** ^1^Department of Obstetrics and Gynecology, University of Colorado Anschutz Medical Campus, Aurora, CO, USA; ^2^Mizan-Tepi University Teaching Hospital, Aman, Bench Maji Zone, Ethiopia; ^3^Village Health Partnership, Denver, CO, USA

## Abstract

**Background:**

Primary cesarean birth rates were high among women who were either nulliparous (Group 2) or multiparous (Group 4) with a single, cephalic, term fetus who were induced, augmented, or underwent cesarean birth before labor in our study cohort.

**Objectives:**

The objective of this analysis was to determine what risk factors were associated with cesarean birth among Robson Groups 2 and 4.

**Methods:**

This study was a prospective hospital-based cross-sectional analysis of a convenience sample of 1,000 women who delivered at Mizan-Tepi University Teaching Hospital in the summer and fall of 2019.

**Results:**

Women in Robson Groups 2 and 4 comprised 11.4% (*n* = 113) of the total population (*n* = 993). The cesarean birth rate in Robson Group 2 (*n* = 56) was 37.5% and in Robson Group 4 (*n* = 57) was 24.6%. In Robson Group 2, of all prelabor cesareans (*n* = 5), one birth was elective cesarean by maternal request; the intrapartum cesarean births (*n* = 16) mostly had a maternal or fetal indication (93.8%), with one birth (6.2%) indicated by “failed induction or augmentation,” which was a combined indication. In Robson Group 4, all 4 women delivered by prelabor cesarean had a maternal indication (one was missing data), and 3 of the intrapartum cesareans were indicated by “failed induction or augmentation.” In multivariable modeling of Robson Group 2, having a labor duration of “not applicable” increased the risk of cesarean delivery (RR 2.9, CI (1.5, 5.4)). The odds of requiring maternal antibiotics was the only notable outcome with increased risk (RR 11.1, CI (1.9, 64.9)). In multivariable modeling of Robson Group 4, having a labor longer than 24 hours trended towards a significant association with cesarean (RR 3.6, CI (0.9, 14.3)), and women had a more dilated cervix on admission trended toward having a lower odds of cesarean (RR 0.8, CI (0.6, 1.0)).

**Conclusion:**

Though rates of primary cesarean birth among women who have a term, single, cephalic fetus and are induced, augmented, or undergone prelabor cesarean birth are high, those that occur intrapartum seem to be associated with appropriate risk factors and indications, though we cannot say this definitely as we did not perform an audit. More research is needed on the prelabor subgroup as a separate entity.

## 1. Introduction

The World Health Organization recommends applying the Robson classification for cesarean birth to birth cohorts to better understand which of ten mutually exclusive subgroups are contributing to cesarean birth rates [1, 2]. Analyses generally focus on nulliparous (Group 1) and multiparous (Group 3) women with single, cephalic, term fetuses in spontaneous labor because these women usually account for the greatest proportion of women delivering in any given cohort and can contribute to preventable primary cesarean birth rates [3]. When we applied the Robson classification to a convenience sample of women undergoing cesarean birth at our study site, we found that cesarean birth rates were relatively low in these subgroups (19.4% and 16.1%, respectively, though they accounted for the most cesarean births at the site) [4–7]. We noted that in nulliparous (Group 2) and multiparous (Group 4) women with single, cephalic, term fetuses who required induction, augmentation, or had a cesarean birth prelabor, the cesarean birth rates were very high—37.5% in Group 2 and 24.6% in Group 4. As these groups account for potentially preventable primary cesarean births as well, we wanted to determine risk factors associated with cesarean birth compared to vaginal birth in these subgroups. Our hypothesis was that there might be modifiable risk factors that could be targeted prospectively to reduce unnecessary cesarean births in these subgroups, and our aim was to identify them.

## 2. Materials and Methods

We conducted a hospital-based, prospective, cross-sectional study at Mizan-Tepi University Teaching Hospital (MTUTH), located in Mizan Aman in the Southern Nations, Nationalities, and People's Region (SNNPR). We observed the sample of all women who presented to the facility and gave birth on labor and delivery at MTUTH between May 6 and October 21, 2019, which was the point at which we observed 1,000 births. Women were required to deliver at or after 28 completed weeks of pregnancy to be included in the overall cohort. Women included in Robson Groups 2 and 4 were included in this analysis. Highly trained physicians collected deidentified data through a combination of chart review and structured interview at admission, delivery, and discharge. After data were collected on paper forms and reviewed for completeness, it was entered into REDCap for storage on a password protected server at the University of Colorado, Aurora, Colorado, USA [8].

STATA software version 15.2 (StataCorp LP, College Station, TX, USA) was used for analysis. Bivariate comparisons of sociodemographic, obstetric, birth, and pregnancy outcomes of women experiencing vaginal versus cesarean birth were performed, utilizing Fisher's exact, chi-squared, and Kruskal–Wallis tests depending on the variables. All covariates significant to *p* < 0.05 were included in a multivariable Poisson model with robust error variance (because cesarean birth was prevalent) to determine which covariates were independently associated with cesarean birth. Subsequently, individual logistic regressions (because the outcomes were not as prevalent) of maternal and perinatal outcomes (significant in the bivariate comparisons) were run with the outcomes as the dependent variable and cesarean birth as the independent variable, adjusted for all covariates significant in the multivariable Poisson model, to describe the association between cesarean birth and adverse pregnancy outcomes.

Despite the quality improvement nature of the work and the fact that only deidentified data were collected, oral consent was obtained from each woman before any of her data were recorded. This quality improvement survey was given an exempt from human subjects' research approval (COMIRB # 18–2738) by the University of Colorado, and approval was given by Mizan-Tepi University Teaching Hospital.

## 3. Results

**Figure 1:**
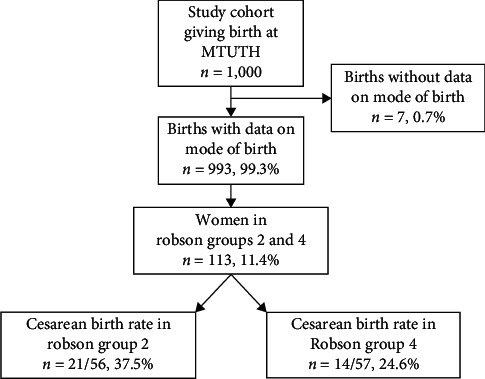
Women giving birth by cesarean in Robson Groups 2 and 4 at Mizan-Tepi University Teaching Hospital.

Our study cohort is defined in Figure 1. Data on mode of delivery were only available in 99.3% of the cohort. When the Robson classification was applied to the 993 women, 113 (11.4%) of them were qualified as being in Robson Groups 2 and 4 (including women with both vaginal and cesarean birth). The population was nearly equally divided between Groups 2 and 4 with 21 (37.5%) of 56 women in Group 2 delivered by cesarean and 14 (24.6%) of 57 women in Group 4 giving birth by cesarean.

Table 1 notes the indications for cesarean births that occurred in these subgroups, divided into those that occurred prior to the onset of labor (prelabor) and those that occurred during the labor course (intrapartum). In Robson Group 2, nulliparous women with a single, cephalic, term fetus who were induced, required augmented, or were delivered before labor, 23.8% (*n* = 5) underwent prelabor cesarean and the remainder (*n* = 16, 76.2%) were delivered during the course of labor. In one of five women delivered prelabor, the indication was clearly identified as cesarean birth by maternal request, with the remaining women delivered for reportedly maternal or fetal indications. Intrapartum cesareans were reportedly performed for the following indications: maternal (37.5%), fetal (56.3%), and failed induction or augmentation of labor (6.2%). Comparatively, among Robson Group 4, all prelabor cesareans (*n* = 4) had a maternal indication (*n* = 1 was missing), while three of the total intrapartum cohort (*n* = 10) underwent cesarean birth for failed induction or augmentation of labor. The remaining intrapartum cesareans in this cohort had a maternal (*n* = 2), fetal (*n* = 4), or combined maternal/fetal indication (*n* = 1).

Table 2 illustrates the bivariate comparisons and multivariable modeling of risk factors associated with cesarean birth as compared to vaginal birth and a description of the Robson Group 2 population. Overall, the cohort is young (median age 21 years), 8.9% illiterate, almost half of Protestant religion, 94.6% are not single, just over half live in an urban setting, and the median number of prenatal visits was 4. Comparing women who had a cesarean birth to those experiencing vaginal birth, labor was more likely to be longer than 24 hours (8.6% versus 19.1%, *p*=0.007), and there was a trend towards significance of more infants < 2500 g being delivered by cesarean (2.9% versus 19.1%, *p*=0.06). Maternal postpartum antibiotic administration (42.9% versus 5.7%, *p*=0.001) was more common after cesarean birth, as was a lower five-minute Apgar score (8 versus 9, *p* < 0.001). Multivariable modeling, which included labor duration and birthweight as covariates, found that only a labor duration of “not applicable” was associated with an increased risk of cesarean when compared to labor less than 12 hours (RR 3.6, *p* < 0.001). In subsequent individual logistic regressions adjusted for labor duration, cesarean birth in Robson Group 2 women was associated with an increased odds of maternal postpartum antibiotic use (OR 11.1, *p*=0.008) but not with Apgar score.

Table 3 provides a similar analysis to Table 2 but for women who are multiparous, term, and have a singleton fetus in cephalic presentation and were induced, augmented, or underwent cesarean birth before labor (Robson Group 4). The population is older than Robson Group 2, with a median age of 27, has a higher rate of illiteracy (21.1%), is also almost half Protestant and mostly not single (98.3%), has just over half of women living in urban settings, and has a median number of 4 prenatal visits. In bivariate comparisons by mode of birth, women undergoing cesarean birth were more likely to live in an urban setting (78.6% versus 46.5%, *p*=0.04) and were more likely to have a labor longer than 24 hours (7.1% versus 2.3%, *p*=0.005), and there was a trend toward a significant difference in their admission cervical dilation (0 cm versus 2 cm, *p*=0.06). Maternal postpartum antibiotic use (28.6% versus 2.3%, *p*=0.003) and lower five-minute Apgar score (8 versus 9, *p*=0.03) were more common after cesarean birth, but macerated stillbirth was less common (0.0% versus 4.7%, *p* < 0.001). In multivariable modeling of the association of these risk factors (including cervical dilation with borderline significance) with cesarean birth in this subgroup, labor duration of longer than 24 hours (RR 3.6, *p*=0.07) and likelihood of cesarean birth with each increasing centimeter of cervical dilation on admission (RR 0.8, *p*=0.07) had borderline significance. Logistic regressions of the association of cesarean birth with pregnancy outcomes did not converge.

## 4. Discussion

Of 113 women at MTUTH who had term, singleton, cephalic fetuses and underwent induced or augmented labor or prelabor cesarean birth, the overall (and primary) cesarean birth rate was 31.0% (data not shown). For nulliparous women in this cohort, the rate was higher at 37.5% and for multiparous women lower at 24.6%. All intrapartum cesarean births in this population reportedly had maternal or fetal indications for cesarean delivery, while at least one of the prelabor surgeries was qualified as cesarean birth by maternal request (though no audit was performed). Across the whole cohort, prolonged duration of labor was associated with cesarean birth. In Group 2, lower birthweight had a trend toward significance, Apgar scores were lower, and maternal antibiotic usage was higher after cesarean delivery. In Group 4, urban living was associated with cesarean birth, and less cervical dilation on admission trended toward significance. Similar to Group 2, Apgar score and maternal antibiotic usage were higher after cesarean birth in bivariate comparisons but unable to be assessed by multivariable modeling given the small sample size.

Prolonged duration of labor stands out as an important predictor of cesarean birth in Robson Groups 2 and 4. Traditional obstetric teaching suggests that nulliparous women should deliver within 20 hours of the onset of labor and multiparous women with 14 hours [9]. More recent literature has suggested that rather than a strict time-based assessment of progress in labor, other obstetric indicators may signal what is referred to as prolonged, dysfunctional, protracted, and/or obstructed labor [10, 11]. To assist labor and delivery providers with making these determinations, there is the partogram, intrapartum decision-making support tools, and guidelines on preventing primary cesarean birth [12].

The partogram is currently in use at MTUTH but has variable levels of completion, which would be an area for quality improvement at the facility. While prior Cochrane reviews have questioned the association of partogram use with improved pregnancy outcomes, more recent literature has suggested that use of the tool as intended is associated with the decision to proceed to cesarean birth and reduced stillbirth [13]. An audit and feedback assessment of partogram use on the labor floor might give the MTUTH team a better sense of how, when, for whom, and at what quality level the partograms are being completed. Anecdotal reports from the site have indicated that the partogram is often completed retrospectively only to ensure that the medical record is complete, which, if true, would preclude the tool from assisting with intrapartum decision making or monitoring of labor progress.

Regarding intrapartum decision-making support tools, there are applications designed for smartphones that assist providers with interpreting fetal heart rate monitoring and the partogram [14, 15]. If initial audit and feedback indicates that the partogram is not being used properly at MTUTH or that providers are having difficulty understanding, completing, or interpreting the partogram, considering the use of intrapartum management tools might be an option to facilitate improved labor management and assist providers with clinical decision making. Prior research from Ethiopia has shown that medical doctors and higher level clinicians, workers at health centers, and providers exposed to in-service trainings had a higher adjusted odds of being willing to use an “e-partograph” [15]. Surveying the availability of smartphones among providers at MTUTH and piloting the use of this tool while tracking process, implementation, and health outcomes measures would be a contribution to the literature. Additionally, some work has been performed in Tanzania to evaluate the effect of locally tailored labor management guidelines on fetal outcomes, which could provide some additional guidance on quality improvement interventions [16].

Despite the recognition that obstructed labor, of which duration of labor is a proxy measure, is an appropriate indication for cesarean birth, as stated previously, time alone may not be a rich enough variable to support the decision to move to cesarean. Fortunately, organizations in other countries have introduced guidelines for prevention of primary cesarean birth that focus on additional specific measures of labor and delivery progress [10]. Adherence to the guidelines can assist a provider with determining when a trial of labor, induction of labor, or augmentation of labor has failed, based on evidence. Though prior research has shown that recommendations from high-income settings do not always translate and may not always be appropriate for lower-income sites, MTUTH may consider a quality improvement initiative to review and adapt published guidelines involving relevant stakeholders in that modification process. A trial of strict enforcement of the guidelines in a subset of women may give some indication as to how these guidelines assist with labor management at MTUTH and how they may lower or raise the cesarean birth rate among these and other Robson subgroups.

Robson Groups 2 and 4 have a higher primary cesarean birth rate (31.0%) than that of Robson Groups 1 and 3 (17.6%, data no shown), the latter of which are known to contribute most significantly to primary cesarean birth rates, globally [3]. The difference between Groups 2 and 4 versus Groups 1 and 3 is that the former goes into labor spontaneously and delivers without augmentation and the latter requires induction, augmentation, or was delivered by cesarean before the onset of labor. It may be the onset of labor or lack of augmentation that mostly accounts for the drastic difference in cesarean birth rates between these groups, although we are unable to test that association as the variable is used to define the groups themselves. While some data have shown that induction may be associated with cesarean birth (depending on indication), a recent large, randomized trial of nulliparous, singleton, term women with a cephalic fetus showed that women who were induced (a subset of Robson Group 2) had a lower cesarean birth rate than the control group who underwent usual care [17, 18]. This is why a further separation of Groups 2 and 4 into those cesareans that occur prelabor, those that are induced, and those that are augmented would be important, as other authors have noted [19].

The limitations of our study are the small sample size of the subgroups of interest and the lack of audit to confirm indication for cesarean birth. Additionally, we were unable to calculate a logistic regression for Group 4 due to the small sample. Strengths of our study include the collection of a comprehensive set of covariates and outcome measures to allow for good hypothesis generation.

## 5. Conclusion

In conclusion, we have not determined with this analysis what accounts for the higher cesarean birth rate among Robson Groups 2 and 4, except for the fact that some cesareans are performed before the onset of labor. There is not enough information regarding the circumstances of women undergoing prelabor cesarean birth at MTUTH in this cohort beyond very general indications, which would be a great area for further evaluation. We do know that one woman was granted cesarean birth by maternal request, and it will be important to watch the trend in this practice over time to determine if it is becoming more common, which the World Health Organization does not recommend [20–22]. We were unable to identify any national or international guidelines on appropriate, evidence-based indications for prelabor cesarean birth, and we think this is an important gap in knowledge that needs to be addressed.

## Figures and Tables

**Table 1:** Cesarean birth by indication in Robson Groups 2 and 4 at Mizan-Tepi University Teaching Hospital.

Indications for cesarean birth in Robson Group 2 (*n* = 21, 37.5%)		
	Prelabor cesarean (*n* = 5, 23.8%)	Intrapartum cesarean (*n* = 16, 76.2%)
Elective cesarean birth	1 (20.0%)	NA
Maternal indication only	2 (40.0%)	6 (%)
Fetal indication only	2 (40.0%)	9 (%)
Failed induction/augmentation	NA	1 (%)
Missing	NA	NA

Indications for cesarean birth in Robson Group 4 (*n* = 14, 24.6%)		
	Prelabor cesarean (*n* = 4, 28.6%)	Intrapartum cesarean (*n* = 10, 71.4%)
Maternal indication only	3 (75.0%)	2 (%)
Fetal indication only	NA	4 (%)
Failed induction/Augmentation	NA	3 (%)
Maternal and fetal indication	NA	1 (%)
Missing	1 (25.0%)	NA

**Table 2:** Sociodemographic, obstetric, labor, and delivery characteristics of Robson Group 2 women who experienced cesarean birth compared to vaginal birth, bivariate comparisons, and multivariable models.

Table 2A. Bivariate comparisons				
Characteristic	Robson group 2 overall (*n* = 56)	Robson group 2 vaginal birth (*n* = 35, 62.5%)	Robson group 2 cesarean birth (*n* = 21, 37.5%)	*p* value
Sociodemographic				
Age in years, median (IQR)	21 (20, 24)	22 (20, 24)	20 (20, 24)	0.79^a^
Missing	0 (0.0%)	0 (0.0%)	0 (0.0%)	

Education				0.49^b^
Unable to read and write	5 (8.9%)	4 (11.4%)	1 (4.8%)	
Read and write only	2 (3.6%)	0 (0.0%)	2 (9.5%)	
Primary school	23 (41.1%)	14 (40.0%)	9 (42.9%)	
Secondary school	9 (16.1%)	6 (17.1%)	3 (14.3%)	
Higher education	17 (30.4%)	11 (31.4%)	6 (28.6%)	
Missing	0 (0.0%)	0 (0.0%)	0 (0.0%)	

Religion				0.23^b^
Muslim	4 (7.1%)	1 (2.9%)	3 (14.3%)	
Orthodox Christian	25 (44.6%)	15 (42.9%)	10 (47.6%)	
Catholic Christian	0 (0.0%)	0 (0.0%)	0 (0.0%)	
Protestant	27 (48.2%)	19 (54.3%)	8 (38.1%)	
Jehovah's witness	0 (0.0%)	1 (0.4%)	0 (0.0%)	
Missing	0 (0.0%)	1 (0.4%)	0 (0.0%)	

Relationship status				1.0^b^
Single	2 (3.6%)	2 (5.7%)	0 (0.0%)	
Not single	53 (94.6%)	33 (94.3%)	20 (95.2%)	
Missing	1 (1.8%)	0 (0.0%)	1 (4.8%)	

Woreda				0.53^c^
Urban	29 (51.8%)	17 (48.6%)	12 (57.1%)	
Rural	27 (48.2%)	18 (51.4%)	9 (42.9%)	
Missing	0 (0.0%)	0 (0.0%)	0 (0.0%)	

Number of prenatal visits				0.69^a^
Median (IQR)	4 (3, 5)	4 (3, 5)	4 (4, 5)]	

Antepartum, labor, and delivery				
Transferred during labor				0.53^c^
No	27 (48.2%)	18 (51.4%)	9 (42.9%)	
Yes	29 (51.8%)	17 (48.6%)	12 (57.1%)	
Missing	0 (0.0%)	0 (0.0%)	0 (0.0%)	

Cervical exam on admission				0.35^a^
Median (IQR)	2 (0, 3)	3 (1, 4)	2 (0, 3)	

Duration of labor				0.007^b^
Not applicable	5 (8.9%)	0 (0.0%)	5 (23.8%)	
<12 hours	26 (46.4%)	18 (51.4%)	8 (38.1%)	
12–24 hours	18 (32.1%)	14 (40.0%)	4 (19.1%)	
24+ hours	7 (12.5%)	3 (8.6%)	4 (19.1%)	
Missing	0 (0.0%)	0 (0.0%)	0 (0.0%)	

Antepartum hemorrhage				0.55^b^
No	53 (94.6%)	34 (97.1%)	19 (90.5%)	
Yes	3 (5.4%)	1 (2.9%)	2 (9.5%)	
Missing	0 (0.0%)	0 (0.0%)	0 (0.0%)	

Antepartum preeclampsia/eclampsia/chronic hypertension				0.15^b^
No	46 (82.1%)	31 (88.6%)	15 (71.4%)	
Yes	10 (17.9%)	4 (11.4%)	6 (28.6%)	
Missing	0 (0.0%)	0 (0.4%)	0 (0.0%)	

Birthweight (grams)				0.06^c^
<2500	5 (8.9%)	1 (2.9%)	4 (19.1%)	
≥ 2500	51 (91.1%)	34 (97.1%)	17 (80.9%)	
Missing	0 (0.0%)	0 (0.0%)	0 (0.0%)	

Postpartum complications				
MATERNAL				

Postpartum blood transfusion				0.38^b^
No	55 (98.2%)	35 (100.0%)	20 (95.2%)	
Yes	1 (1.8%)	0 (0.0%)	1 (4.8%)	
Missing	0 (0.0%)	0 (0.0%)	0 (0.0%)	

Postpartum antibiotics				0.001^b^
No	45 (80.4%)	33 (94.3%)	12 (57.1%)	
Yes	11 (19.6%)	2 (5.7%)	9 (42.9%)	
Missing	0 (1.0%)	0 (0.0%)	0 (5.2%)	

Postpartum hypertensive treatment				0.35^b^
No	51 (91.1%)	33 (94.3%)	18 (85.7%)	
Yes	5 (8.9%)	2 (5.7%)	3 (14.3%)	
Missing	0 (1.0%)	0 (0.0%)	0 (5.2%)	

NEONATAL				

Five-minute Apgar score median (IQR)	9 (8, 9)	9 (8, 9)	8 (7, 8)	<0.001^a^
Missing	0 (0.0%)	0 (0.0%)	0 (0.0%)	

Stillbirth				0.38^b^
Yes, fresh	1 (1.8%)	0 (0.0%)	1 (4.8%)	
Yes, macerated	0 (0.0%)	0 (0.0%)	0 (0.0%)	
No	55 (98.2%)	35 (100.0%)	20 (95.2%)	
Missing	0 (0.0%)	0 (0.0%)	0 (0.0%)	

Antibiotics				1.0^b^
No	55 (98.2%)	34 (97.1%)	21 (100.0%)	
Yes	1 (1.8%)	1 (2.9%)	0 (0.0%)	
Missing	0 (0.0%)	0 (0.0%)	0 (0.0%)	

Neonate status on day of discharge				0.55^b^
Dead	3 (5.4%)	1 (2.9%)	2 (9.5%)	
Alive	53 (94.6%)	34 (97.1%)	19 (90.5%)	
Missing	0 (0.3%)	35 (0.4%)	0 (0.0%)	

Table 2B. Multivariable model of characteristics associated with cesarean birth and association of cesarean birth with pregnancy outcomes				

2B1. Multivariable Poisson model with robust error variance of characteristics associated with cesarean birth				

Characteristic	RR	CI	*p* value	

Compared to less than 12 hours of labor				
Not applicable	2.9	1.5, 5.4	0.001	

2B2. Individual logistic regressions, adjusted for significant findings in (2B1) to determine association of cesarean birth (CB) with outcomes significant in bivariate comparisons (2A)				

Maternal outcomes				

	OR	CI	*p* value	

Odds of requiring postpartum antibiotics after CB	11.1	1.9, 64.9	0.008	

Neonatal outcomes				

	OR	CI	*p* value	

Odds of having a higher Apgar score after CB	0.75	0.04, 15.2	0.85	

Kruskal–Wallis test. ^b^Fisher's Exact test. ^c^chi-squared test.

**Table 3:** Sociodemographic, obstetric, labor, and delivery characteristics of Robson Group 3 women who experienced cesarean birth compared to vaginal birth, bivariate comparisons, and multivariable models.

(3A) Bivariate comparisons				
Characteristic	Robson Group 4	Robson Group 4	Robson Group 4	*p* value
	Overall (*n* = 57)	Vaginal birth (*n* = 43, 75.4%)	Cesarean birth (*n* = 14, 24.6%)	
Sociodemographic				
Age in years, median (IQR)	27 (24, 30)	27 (25, 30)	26 (20, 30)	0.28^a^
Missing	1 (0.3%)	1 (0.3%)	0 (0.0%)	

Education				0.26^b^
Unable to read and write	12 (21.1%)	10 (23.3%)	2 (14.3%)	
Read and write only	2 (3.5%)	2 (4.7%)	0 (0.0%)	
Primary school	22 (38.6%)	17 (39.5%)	5 (35.7%)	
Secondary school	9 (15.8%)	4 (9.3%)	5 (35.7%)	
Higher education	12 (21.1%)	10 (23.3%)	2 (14.3%)	
Missing	0 (0.0%)	0 (0.0%)	0 (0.0%)	

Religion				0.32^b^
Muslim	11 (19.3%)	9 (20.9%)	2 (14.3%)	
Orthodox Christian	20 (35.1%)	17 (39.5%)	3 (21.4%)	
Catholic Christian	0 (0.0%)	0 (0.0%)	0 (0.0%)	
Protestant	26 (45.6%)	17 (39.5%)	9 (64.3%)	
Jehovah's witness	0 (0.0%)	0 (0.0%)	0 (0.0%)	
Missing	0 (0.0%)	0 (0.0%)	0 (0.0%)	

Relationship status				1.0^b^
Single	0 (0.0%)	0 (0.0%)	0 (0.0%)	
Not single	56 (98.3%)	42 (97.7%)	14 (100.0%)	
Missing	1 (1.8%)	1 (2.3%)	0 (0.0%)	

Woreda				0.04^c^
Urban	31 (54.4%)	20 (46.5%)	11 (78.6%)	
Rural	26 (45.6%)	23 (53.5%)	3 (21.4%)	
Missing	0 (0.0%)	0 (0.0%)	0 (0.0%)	

Number of prenatal visits				0.83^a^
Median (IQR)	4 (4, 5)	4 (4, 5)	4 (3, 6)	

Antepartum, labor, and delivery				

Transferred during labor				0.49^c^
No	33 (57.9%)	26 (60.5%)	7 (50.0%)	
Yes	24 (42.1%)	17 (39.5%)	7 (50.0%)	
Missing	0 (0.0%)	0 (0.0%)	0 (0.0%)	

Cervical exam on admission				0.06^a^
Median (IQR)	2 (0, 3)	2 (0, 3)	0 (0, 2)	

Duration of labor				0.005^b^
Not applicable	7 (12.3%)	2 (4.7%)	5 (35.7%)	
<12 hours	28 (49.1%)	25 (58.1%)	3 (21.4%)	
12–24 hours	20 (35.1%)	15 (34.9%)	5 (35.7%)	
24+ hours	2 (3.5%)	1 (2.3%)	1 (7.1%)	
Missing	0 (0.0%)	0 (0.0%)	0 (0.0%)	

Antepartum hemorrhage				0.43^b^
No	55 (96.5%)	42 (97.7%)	13 (92.9%)	
Yes	2 (3.5%)	1 (2.3%)	1 (7.1%)	
Missing	0 (0.0%)	0 (0.0%)	0 (0.0%)	

Antepartum preeclampsia/eclampsia/chronic hypertension				0.32^b^
No	52 (91.2%)	38 (88.4%)	14 (92.9%)	
Yes	5 (8.8%)	5 (11.6%)	0 (7.1%)	
Missing	0 (0.0%)	0 (0.0%)	0 (0.0%)	

Birthweight (grams)				
<2500	4 (7.0%)	3 (7.0%)	1 (7.1%)	1.0^b^
≥ 2500	53 (93.0%)	40 (93.0%)	13 (92.9%)	
Missing	0 (0.0%)	0 (0.0%)	0 (0.0%)	
Postpartum complications				

MATERNAL				

Postpartum hemorrhage				1.0^b^
No	56 (98.3%)	42 (97.7%)	14 (100.0%)	
Yes	1 (1.7%)	1 (2.3%)	0 (0.0%)	
Missing	0 (0.0%)	0 (0.0%)	0 (0.0%)	

Postpartum blood transfusion				0.25^b^
No	56 (98.3%)	43 (100.0%)	13 (92.9%)	
Yes	1 (1.7%)	0 (0.0%)	1 (7.1%)	
Missing	0 (0.0%)	0 (0.0%)	0 (0.0%)	

Postpartum antibiotics	52 (91.2%)	42 (97.7%)	10 (71.4%)	0.003^c^
No	5 (8.8%)	1 (2.3%)	4 (28.6%)	
Yes	0 (0.0%)	0 (0.0%)	0 (0.0%)	
Missing				

Postpartum hypertensive treatment				0.57^b^
No	54 (94.7%)	40 (93.0%)	14 (100.0%)	
Yes	3 (5.3%)	3 (7.0%)	0 (0.0%)	
Missing	0 (0.0%)	0 (0.0%)	0 (0.0%)	

NEONATAL				

Five-minute Apgar score median (IQR)	9 (8, 9)	9 (8, 9)	8 (8, 9)	0.03^a^
Missing	0 (0.0%)	0 (0.0%)	0 (0.0%)	
Stillbirth	0 (0.0%)	0 (0.0%)	0 (0.0%)	<0.001^b^
Yes, fresh	2 (3.5%)	2 (4.7%)	0 (0.0%)	
Yes, macerated	55 (96.5%)	41 (95.4%)	14 (100.0%)	
No	0 (0.0%)	0 (0.0%)	0 (0.0%)	
Missing				

Neonate status on day of discharge	2 (3.5%)	2 (4.7%)	0 (0.0%)	1.0^b^
Dead	54 (94.7%)	40 (93.0%)	14 (100.0%)	
Alive	1 (1.8%)	1 (2.3%)	0 (0.0%)	
Missing				

(3B) Multivariable model of characteristics associated with cesarean birth				

Multivariable Poisson model with robust error variance of characteristics associated with cesarean birth				

Characteristic	RR	CI	*p* value	

Compared to less than 12 hours of labor	2.8	0.7, 12.0	2.0	
Likelihood of cesarean if labor duration “not applicable”	0.2	0.5, 8.1	0.3	
Likelihood of cesarean if labor duration 12–24 hours	3.6	0.9, 14.3	0.07	
Likelihood of cesarean if labor duration >24 hours				

Likelihood of cesarean of rural compared to urban location	0.6	0.1, 2.4	0.4	

Likelihood of cesarean with each increasing centimeter of dilation on admission cervical exam	0.8	0.6, 1.0	0.07	

^a^Kruskal–Wallis test. ^b^Fisher's Exact test. ^c^Chi-squared test. ^d^Variables included in the model without an association with the outcome: urban/rural residence, diagnosis of chorioamnionitis, and infant birthweight.

## Data Availability

The data used to support this study are made available from the corresponding author upon request.

## Abbreviations

MTUTH: Mizan-Tepi University Teaching Hospital
SNNPR: Southern Nations, Nationalities, and People's Region.
